# Surgical outcome of epicanthus and telecanthus correction by C-U medial canthoplasty with lateral canthoplasty in treatment of Blepharophimosis syndrome

**DOI:** 10.1186/s12886-022-02455-2

**Published:** 2022-05-19

**Authors:** Ahmed Ali Amer, Marwa Mahmoud Abdellah, Nader Hussein Fouad Hassan, Amr Mounir

**Affiliations:** 1 Ophthalmology Department Qena Faculty of medicine, South Valley University, Qena, Egypt; 2 Sohag Faculty of Medicine, Ophthalmology Department, Sohag University, Sohag City, Egypt; 3 Benha Faculty of Medicine, Ophthalmology Department, Benha University, Benha, Egypt

**Keywords:** Epicanthus, Telecanthus, C-U medial canthoplasty, lateral canthoplasty, Blepharophimosis Syndrome

## Abstract

**Purpose:**

To evaluate the surgical outcome of epicanthus and telecanthus correction by C-U medial canthoplasty with lateral canthoplasty in Blepharophimosis Syndrome.

**Patients and methods:**

This was a retrospective single arm interventional study including 18 eyes of 9 patients with Blepharophimosis-ptosis-epicanthus inversus syndrome who presented to oculoplastic clinic, ophthalmology department, Qena university hospital in the period of between July 2020 to April 2021. All the patients had BPES with epicanthus and telecanthus. All cases were subjected to by C plasty with medial and lateral canthoplasty for correction of epicanthus and telecanthus correction followed by frontalis suspension surgery to correct the co-existing blepharoptosis.

**Results:**

The study included 9 cases of BPES, 6 boys and 3 girls, the mean age was 5.4 ± 1.5 in the study group, all patients had a positive family history for BPES. After surgery, the mean IICD decreased from 38.44 mm preoperatively to 32.8 mm postoperatively, with a mean difference of 6.2 mm (*P* <  0.001). Likewise, the mean PFL increased from 20.78 mm preoperatively to 26.63 mm postoperatively, with a mean difference of 5.8 mm (*P* <  0.001). Epicanthus skin fold disappeared in all cases and medical canthus could be seen with well healed difficulty seen scars.

**Conclusion:**

C-U medial canthoplasty with lateral canthoplasty in Blepharophimosis Syndrome was found to be an effective procedure in the correction of epicanthus and telecanthus.

## Introduction

Blepharophimosis-ptosis-epicanthus inversus syndrome (BPES) is inherited developmental anomaly manifested by very narrow horizontal palpebral aperture, epicanthus inversus, blepharoptosis, and telecanthus. It is an uncommon anomaly which inherited as autosomal dominant trait and may be sporadic [1, 2].

Blepharoptosis is due to impaired function or absence of the levator palpebrae muscle which usually bilateral and symmetrical in these patients. Epicanthus inversus is characterized by prolongation of the medial lower eyelid running inward and upward. It is uncommon to improve spontaneously with normal facial development so it is different from other forms of epicanthus. Telecanthus means that there is a lateral displacement of the medial canthal tendon leading to an abnormally large intercanthal distance, with a normal interpupillary distance (IPD) [2, 3].

Many surgical procedures have been used for medial epicanthoplasty in cases of BPES to correct the epicanthus inversus and associated telecanthus including Y-to-V flaps, Blair et al. [4] technique and its modification by Johnson [5].

The Mustardé technique [6] that is the most common technique used for medial epicanthoplasty in patients with BPES. However, this technique necessitates delicate measurements and mapping out of lines and angles based on geometric basis which are complicated and confusing [7, 8].

Also, there is difficulty of intraoperative transposition of the different flaps with flap trimming before suturing [9].

The aim of our study was to evaluate the surgical outcome of epicanthus and telecanthus correction by C- U medial canthoplasty with lateral canthoplasty in Blepharophimosis Syndrome.

## Patients and methods

This was a retrospective single arm interventional study including 18 eyes of 9 patients with Blepharophimosis-ptosis-epicanthus inversus syndrome who presented to oculoplastic clinic, ophthalmology department, Qena university hospital in the period of between July 2020 to April 2021.

All patients involved in this study were informed about the aim of the study and the guardians had signed the informed consent. Our study adhered to the Tenants of Helsinki and the ethical board committee approval of our institution (Qena Faculty of Medicine) was obtained under IBR Registration number: SVU/Med/OPH026/4/22/1/318.

All the patients had BPES with epicanthus and telecanthus. Inclusion criteria of this study were limited to age between 4 and 7 years, all patients with all the components of BPES with ptosis. Exclusion criteria include patients with vertical squint, affected Bell’s phenomenon, Marcus-Gunn jaw winking phenomenon, mild ptosis, previous eyelid surgery, nystagmus, and corneal anesthesia.

One stage reconstructive procedure was used to correct the Telecanthus and epicanthus components of BPES. All patients underwent a full ophthalmological evaluation including visual acuity, ocular motility, slit lamp examination and fundus examination. Eyelid measurements were performed pre and postoperatively and included the Inner Intercanthal Distance (IICD), Horizontal Palpebral Fissure Length (HPFL) and The Marginal Reflex Distance1 (MRD-1). The HPFL was measured from the point of contact of the upper and lower lid medially to the lateral canthus. IICD was measured from the point of contact of the upper lid with the lower lid on both sides.

All cases were followed up for 12 months post-surgery to evaluate the results.

### Surgical procedure

All surgeries were done under general anesthesia. Firstly, amount of correction of the telecanthus is determined depending on that the ideal intercanthal distance should be at least half of the interpupillary distance. Due to the postoperative fibrosis at site of surgery, we targeted that the amount of tissue excised has to be at least 1.5 times of the amount of desired correction. So, amount of telecanthus correction needed on each eye = ½ (preoperative IICD-IPD). e.g. in a patient with IICD 38 mm and IPD 56 mm amount of telecanthus correction for each eye = ½ (38–28) = 5 mm for each eye.

**Fig. 1:**
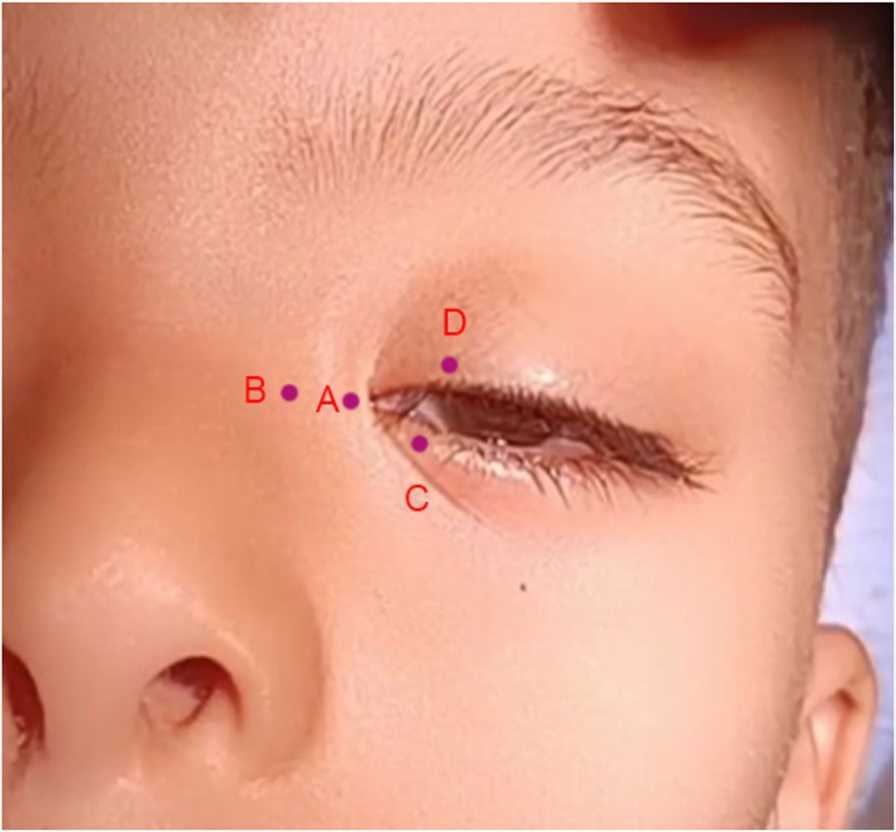
Four points (**A**-**D**) drawn to determine the C and U-shaped incisions

Four points were drawn to determine the C and U-shaped incisions. Point A is located at 2 mm medial to the existing medial canthus and point B is located medial to point A to a distance equal to the amount of tissue to be excised to obtain the desired correction of telecanthus. Then the upper and lower extend of the C shaped incision is marked passing by point C which is located at the level of lower punctum 2 mm from the lid margin and point D which is located 3 mm lateral to the upper punctum and 2 mm from the lid margin so that a small curvilinear line is drawn joining points C, A and D forming the C limb of medical canthoplasty. Another small curvilinear line is drawn joining points C, B and D forming the U limb of medical canthoplasty. (Fig. 1).

Amount of tissue needed to be excised in each eye = amount of desired correction Χ 1.5 e.g. in this patient amount of tissue to be excised = 5 Χ 1.5 = 7.5 mm (this is the distance between points A and B).

The skin and muscle tissues between the medial and lateral limbs is excised with exposure of the medial canthal tendon which probing of the lower lacrimal canalicular system to save. Then the medial canthal tendon is plicated and fixed to the periosteum of anterior lacrimal crest using 4/0 prolene suture leading to shortening of the medial canthal tendon and lengthening of the horizontal palpebral fissure length HPFL.

Orbicularis muscle is closed by 6/0 vicryl interrupted sutures and then skin is closed by 6/0 prolene or in some cases 6/0 vicryl interrupted sutures. Lateral canthoplasty is done to lengthen HPFL and achieved by skin incision 5 mm from the existing lateral canthus with lateral canthotomy. Then, the conjunctiva at the lateral canthus is undermined and sutured to the new lateral canthal angle to achieve the sharpness of this angle. The upper and lower edges of the conjunctiva are sutured to extending skin of upper and lower eyelids using interrupted 6/0 vicryl. At least 6 months later, the frontalis suspension surgery was done to correct the co-existing blepharoptosis.

**Fig. 2:**
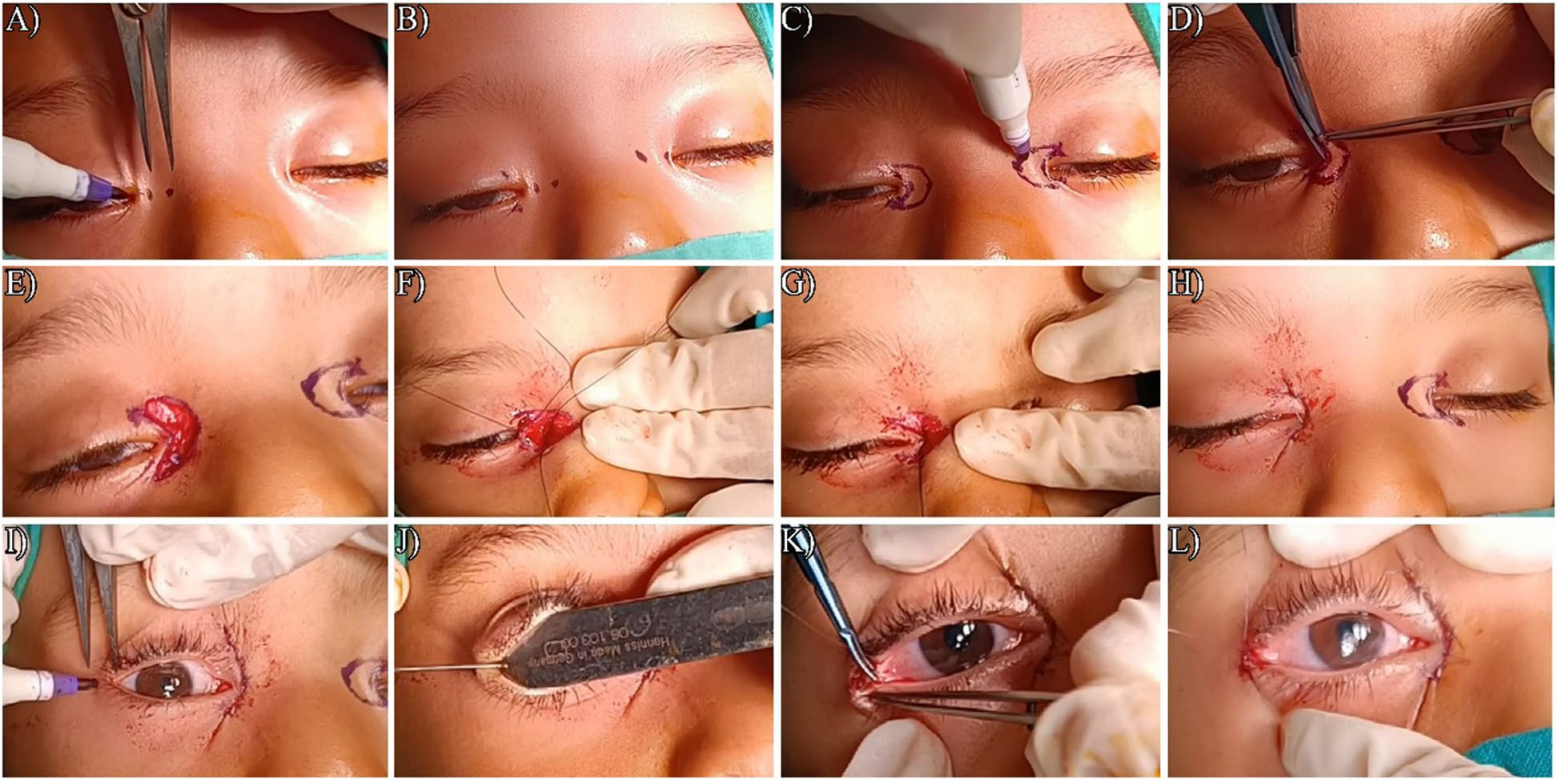
Steps of surgery from A to P for epicanthus and telecanthus correction by C-U with medial canthoplasty and lateral canthoplasty. **A** Marking of points A and B in right eye, **B** Marking of points **C** and **D** right eye and points **A**, **B** in left eye, **C** Marking of the C and U arms of C-U medial canthoplasty in Left eye, **D** excision of the skin and orbicularis muscle in between the C and U arms of the C-U medial canthoplasty, **E** exposure of the medial canthal tendon, **F** Plication of the medial canthal tendon, **G** Shortening of the medial canthal tendon with lengthening HPFL, **H** Complete of the medial wound closure, **I** Marking of the skin for lateral canthoplasty, **J** Lateral canthal wound to lengthen the HPFL, **K **Suturing the undermined conjunctiva to the new lateral canthal angle, **L** making the sharp lateral canthal angle

Steps of surgery were summarized in Fig. 2.

### Statistical analysis

Data were verified, coded by the researcher, and analyzed using IBM-SPSS 24.0 (IBM-SPSS Inc., Chicago, IL, USA) *. Descriptive statistics: Means, standard deviations, median and percentages were calculated. Test of significances: one-way repeated measure ANOVA (RM-ANOVA) test was calculated to test the mean differences of the data that follow normal distribution and had repeated measures, post-hoc test was calculated using Bonferroni corrections for pairwise comparisons between the study groups. A significant *p*-value was considered when it is less than 0.05.

## Results

The study included 9 cases of BPES, 6 boys and 3 girls, the mean age was 5.4 ± 1.5 in the study group, all patients had a positive family history for BPES [Table 1].

**Table 1:** General Information for Patients With BPES

BPES	Male/Female	Family History	Age (Years) Mean ± SD
	**6/3**	**100%**	**5.4 ± 1.5**
**IPD (mm)**	**Mean ± SD**	**55.44 ± 1.2**	
	**Median (Range)**	**55 (53–56)**	

The preoperative IPD mean was 55.4 ± 1.2 with a median of 55 and a range of 53–56 mm [Table 1]. Preoperative IICD ranged from 38 mm to 42 mm. Postoperative IICD at the 1st week ranged from 26 to 31 mm, after 6 months ranged from 30 to 32 mm and after 1 year ranged from 31.5 to 33 mm after correction.

Likewise, the preoperative HPFL was 20.78 ± 1.0 mm. At 1 week postoperatively it was 28.00 ± 0.7 mm, after 6 months was 26.78 ± 0.9 mm and after 1 year was 26.63 ± 0.8 mm after correction. Moreover, preoperative IICD/IPD ratio ranged between 0.69 and 0.75. At 1 week postoperatively it ranged from 0.47 to 0.57, after 6 months ranged from 0.55 to 0.6 and after 1 year ranged from 0.57 to 0.62 after correction. Furthermore, preoperative IICD/HPFL ratio ranged between 1.68 and 2.05. At 1 week postoperatively it ranged from 0.93 to 1.15, after 6 months ranged from 1.09 to 1.23 and after 1 year ranged from 1.14 to 1.29 after correction of telecanthus [Table 2].

**Table 2:** Pre- and Postoperative Measurement of BPES

Patient No.	Visits	IICD mm	IPD mm	HPFL mm	IICD\IPD ratio	IICD\HPFL ratio
**1**	**Pre**	38	55	20	0.69	1.90
	**1 year post**	31.5		25.6	0.57	1.23
**2**	**Pre**	39	53	19	0.74	2.05
	**1 year post**	32		26	0.60	1.23
**3**	**Pre**	42	55	21	0.76	2.00
	**1 year post**	33		26.6	0.60	1.24
**4**	**Pre**	40	53	22	0.75	1.82
	**1 year post**	32		28	0.60	1.14
**5**	**Pre**	37	55	22	0.67	1.68
	**1 year post**	31		26.5	0.56	1.17
**6**	**Pre**	41	56	20	0.73	2.05
	**1 year post**	32		27.5	0.57	1.16
**7**	**Pre**	38	53	21	0.72	1.81
	**1 year post**	33		27.5	0.62	1.20
**8**	**Pre**	40	54	21	0.74	1.90
	**1 year post**	33		25.5	0.61	1.29
**9**	**Pre**	40	56	21	0.71	1.90
	**1 year post**	33		26.5	0.59	1.25

*IICD* inner inter = canthal distance, *IPD* interpupillary distance, *HPFL* horizontal palpebral fissure length

**Fig. 3:**
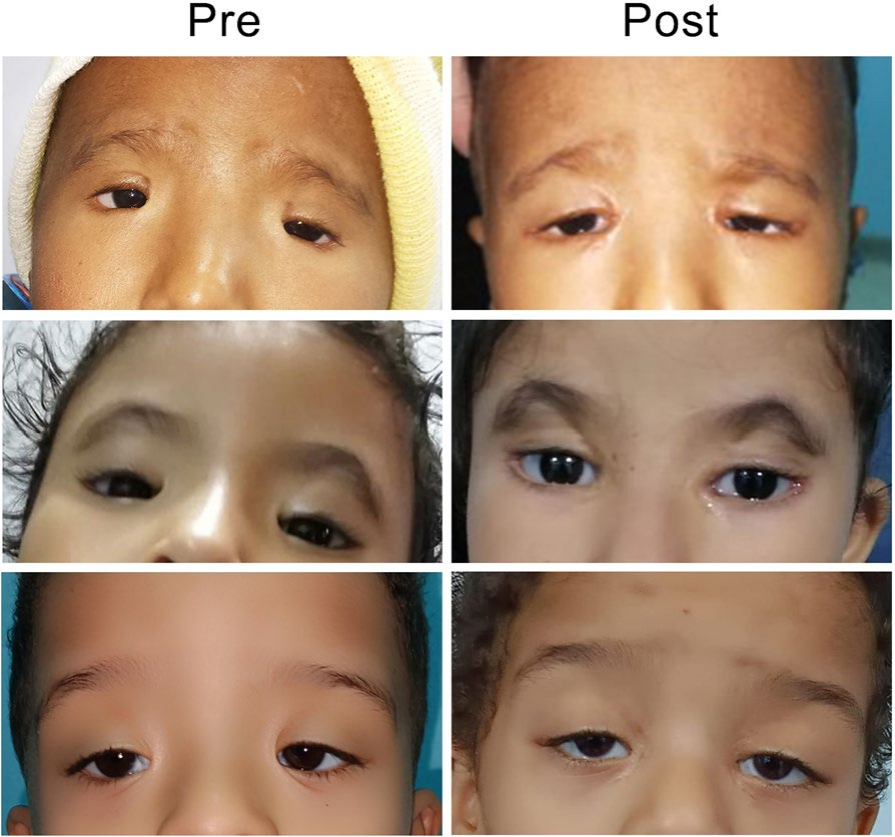
3 cases of BPES before and after one year of surgery

Figure 3 shows 3 cases of BPES before and after 1 year of surgery.

**Fig. 4:**
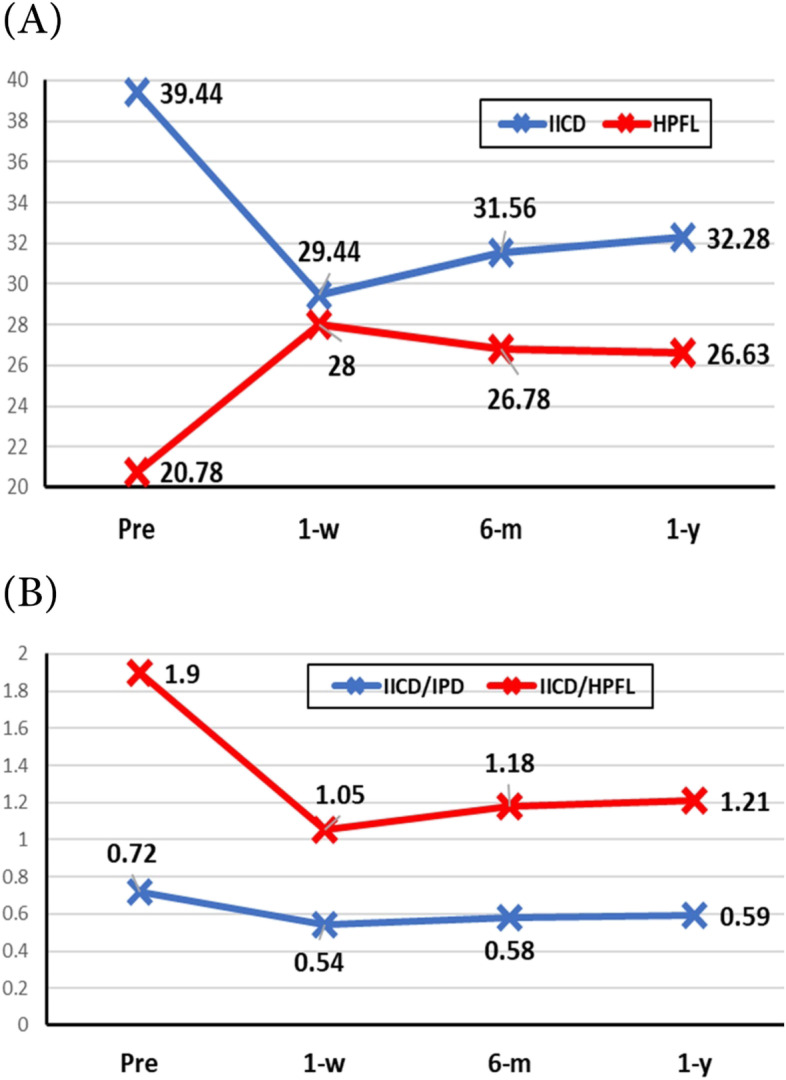
**A** Changes in mean IICD and mean PFL within one year follow up. **B** Changes in the IICD/IPD ratio and the IICD/HPFL ratio within year follow up

Table 3 illustrated the effect of surgery on the studied parameters. After consecutive surgeries, the mean IICD decreased from 39.44 mm preoperatively to 32.8 mm postoperatively, with a mean difference of 6.2 mm (*P* <  0.001). Likewise, the mean PFL increased from 20.78 mm preoperatively to 26.63 mm postoperatively, with a mean difference of 5.8 mm (*P* <  0.001) (Fig. 4 A).

**Table 3:** Preoperative and Postoperative Parameters showing the effect of surgery in studied cases

	Mean ± SD	***P***-value*	***P***-value**	
**IICD (mm)**				
**• Pre-operative**	**39.44 ± 1.6**	**< 0.001**	**1 vs 2 < 0.001**	**2 vs 4 < 0.001**
**• 1-week**	**29.44 ± 1.7**		**1 vs 3 < 0.001**	**3 vs 4 = 0.001**
**• 6-months**	**31.56 ± 0.7**		**1 vs 4 < 0.001**	
**• 1-year**	**32.28 ± 0.8**		**2 vs 3 = 0.002**	
**HPFL (mm)**				
**• Pre-operative**	**20.78 ± 1.0**	**< 0.001**	**1 vs 2 < 0.001**	**2 vs 4 < 0.001**
**• 1-week**	**28.00 ± 0.7**		**1 vs 3 < 0.001**	**3 vs 4 = 0.147**
**• 6-months**	**26.78 ± 0.9**		**1 vs 4 < 0.001**	
**• 1-year**	**26.63 ± 0.8**		**2 vs 3 < 0.001**	
**IICD/IPD Ratio**				
**Pre-operative**	**0.72 ± 0.03**	**< 0.001**	**1 vs 2 < 0.001**	**2 vs 4 < 0.001**
**1-week**	**0.54 ± 0.03**		**1 vs 3 < 0.001**	**3 vs 4 = 0.002**
**6-months**	**0.58 ± 0.02**		**1 vs 4 < 0.001**	
**1-year**	**0.59 ± 0.02**		**2 vs 3 = 0.002**	
**IICD/HPFL Ratio**				
**Pre-operative**	**1.90 ± 0.1**	**< 0.001**	**1 vs 2 < 0.001**	**2 vs 4 < 0.001**
**1-week**	**1.05 ± 0.07**		**1 vs 3 < 0.001**	**3 vs 4 = 0.004**
**6-months**	**1.18 ± 0.05**		**1 vs 4 < 0.001**	
**1-year**	**1.21 ± 0.05**		**2 vs 3 = 0.001**	

^*^One-way RM-ANOVA test was used to compare the mean difference between groups

^**^Post-hoc test was used for pairwise comparison with Bonferroni correction

The IICD/IPD ratio decreased from 0.72 preoperatively to 0.59 postoperatively, with a difference of 0.1 (*P* <  0.001). Likely, the IICD/HPFL ratio decreased from 1.9 preoperatively to 1.21 postoperatively, with a difference of 0.69 (*P* <  0.001) (Fig. 4 B). Regarding epicanthus improvement, the inner canthus could be seen and visualized in all cases with disappearance of the epicanthus fold with well healed scars in all cases. No complications were reported in this study with minimal scaring in all cases.

## Discussion

Congenital Blepharophimosis is an oculofacial maldevelopment syndrome which may occur either as an autosomal dominant trait or sporadically [10]. Most of the known surgical techniques for BPS include multistage procedures. The surgical treatment includes multiple surgical procedures done between 3- and 9-months interval which needs repeated hospitalization and prolonged follow- up [11, 12].

In our study, we evaluated the surgical outcome of epicanthus and telecanthus correction by C-U medial canthoplasty with lateral canthoplasty in blepharophimosis Syndrome. This two-stage reconstructive procedure was used to correct the BPES to avoid multistage procedure for correction. In a study of Keracaoglan et al. [13] had reported one-stage repair of BPS by medial canthoplasty, facial suspension, and widening of the bridge of the nose using the bone graft taken from the iliac crest. However, they had not addressed the combined correction of telecanthus and palpebral phimosis.

In this study, we evaluated success of correction by reduction of telecanthus (IICD) and palpebral phimosis (increasing HPFL) measured preoperatively and through 1 year postoperatively. Cosmetic correction of epicanthus inversus was addressed by appearance of postoperative prominent caruncles.

There was a significant improvement in both mean IICD which decreased from 38.44 mm preoperatively to 32.8 mm postoperatively, with a mean difference of 6.2 mm and the mean PFL which increased from 20.78 mm preoperatively to 26.63 mm postoperatively, with a mean difference of 5.8 mm (*P* < 0.001).

To quantify the severity of epicanthus [14], the ratio of the inner inter-canthal distance (IICD) to interpupillary distance (IPD) (IICD/IPD ratio) was calculated in this study; The IICD/IPD ratio decreased from 0.72 preoperatively to 0.59 postoperatively, with a difference of 0.1 (*P* < 0.001). Also, the IICD/HPFL ratio decreased from 1.9 preoperatively to 1.21 postoperatively, with a difference of 0.69 (*P* < 0.001)**.**

In a study of Wu et al. [15], they performed one-stage correction of BPES, they focused on severity of syndrome and the surgical outcome based on IPFH and the IICD to HPFL ratio as per the normative database in the Chinese population. They found also that medial canthoplasty seems not only to improve the blepharophimosis ratio, but also it was helpful for improving patient appearance.

Another study of Sa HS et al. [16], who described a medial epicanthoplasty technique using the skin redraping method in a retrospective, noncomparative, interventional case series, their results showed that the preoperative median IICD ratio was 1.65 and decreased to 1.27 postoperatively. The median reduction in IICD ratio was 21.7% (*P* < 0.001, Wilcoxon signed-rank test) with conclusion of effectiveness of medial epicanthoplasty using the skin redraping method in the treatment of epicanthus inversus and telecanthus in patients with BPES.

In a study of Bhattacharjee K et al. [17], they evaluated retrospectively the functional and cosmetic outcome of 11 patients of BPS who underwent single-stage surgical correction, they found that single-stage surgical correction of Blepharophimosis syndrome provides stable and successful long-term results.

Another new technique for telecanthus correction were performed by Yamaguchi K et al. [18], who compared between the modified Uchida procedure and the Mustarde procedure on Asian patients with BPES and evaluated the improvement in the basis of preoperative (ICD) ratio (ICD/palpebral fissure width) with favourable and better cosmotic results with the modified Uchida procedure over the Mustarde procedure.

As regards ptosis, patients in this study had no or minimal levator function. Therefore, frontalis suspension was indicated in all patients.

No complications were reported in this study with minimal scaring in all cases with better cosmetic results which favors our results.

In conclusion, C-U medial canthoplasty with lateral canthoplasty with medial and lateral canthoplasty in Blepharophimosis Syndrome was found to be an effective procedure in correction of epicanthus and telecanthus.

## Data Availability

All data generated or analyzed during this study are included in this published article.
